# Social associations in common carp (*Cyprinus carpio*): Insights from induced feeding aggregations for targeted management strategies

**DOI:** 10.1002/ece3.8666

**Published:** 2022-03-07

**Authors:** Peter J. Hundt, Lauren A. White, Meggan E. Craft, Przemyslaw G. Bajer

**Affiliations:** ^1^ 5635 Department of Fisheries, Wildlife, and Conservation Biology University of Minnesota St. Paul Minnesota USA; ^2^ Minnesota Aquatic Invasive Species Research Center (MAISRC) St. Paul Minnesota USA; ^3^ National Socio‐Environmental Synthesis Center University of Maryland Annapolis Maryland USA; ^4^ 5635 Department of Ecology, Evolution and Behavior University of Minnesota St. Paul Minnesota USA

**Keywords:** animal social networks, baiting, contact networks, feeding bouts, invasive species management, social aggregations, social network analysis, spatio‐temporal data streams

## Abstract

Heterogeneity in social interactions can have important consequences for the spread of information and diseases and consequently conservation and invasive species management. Common carp (*Cyprinus carpio)* are a highly social, ubiquitous, and invasive freshwater fish. Management strategies targeting foraging carp may be ideal because laboratory studies have suggested that carp can learn, have individual personalities, a unique diet, and often form large social groups. To examine social feeding behaviors of wild carp, we injected 344 carp with passive integrated transponder (PIT) tags and continuously monitored their feeding behaviors at multiple sites in a natural lake in Minnesota, USA. The high‐resolution, spatio‐temporal data were analyzed using a Gaussian mixture model (GMM). Based on these associations, we analyzed group size, feeding bout duration, and the heterogeneity and connectivity of carp social networks at foraging sites. Wild carp responded quickly to bait, forming aggregations most active from dusk to dawn. During the 2020 baiting period (20 days), 133 unique carp were detected 616,593 times. There was some evidence that feeding at multiple sites was constrained by basin geography, but not distance alone. GMM results suggested that feeding bouts were short, with frequent turnover of small groups. Individual foraging behavior was highly heterogeneous with Gini coefficients of 0.79 in 2020 and 0.66 in 2019. “Superfeeders”—those contributing to 80% of total cumulative detections (top 18% and top 29% of foragers in 2020 and 2019 respectively)—were more likely to be detected earlier at feeding stations, had larger body sizes, and had higher network measures of degree, weighted degree, and betweenness than non‐superfeeders. Overall, our results indicate that wild carp foraging is social, easily induced by bait, dominated by large‐bodied individuals, and potentially predictable, which suggests social behaviors could be leveraged in management of carp, one of the world's most recognizable and invasive fish.

## Graphical abstract

Wild carp responded quickly to bait, forming aggregations most active from dusk to dawn. Individual foraging behavior was heterogeneous, varying in frequency of detections and sites visited. “Superfeeders”—the top 20% most detected individuals—were larger than non‐superfeeders, detected more frequently, and were more socially connected. Wild carp foraging is social, easily induced by bait, dominated by large‐bodied individuals, and potentially predictable, which suggests social behaviors could be leveraged in management of carp, one of the world's most recognizable and invasive fish.

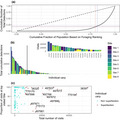

## 1 INTRODUCTION

Social behaviors, including individual personalities, exist across animal taxa (Sih et al., 2004; Wolf & Weissing, 2012) and play a critical role in an organism's evolutionary and ecological history (Kurvers et al., 2014). Social attributes exist along a continuum of behaviors (e.g., bold to shy) and are thought to be static, even when confronted with changes in the environment (Conrad et al., 2011; Sih et al., 2004). Social network analyses of free‐living wildlife have captured this heterogeneity in social interaction, which can have important consequences for the spread of information (Weiss et al., 2014); diseases (Lloyd‐Smith et al., 2005; White et al., 2017); and conservation and/or invasive species management (Fogarty et al., 2011; Haak et al., 2017; Snijders et al., 2017). In particular, one hypothesized phenomenon is the “20/80 rule” which suggests that 20% of individuals are responsible for 80% of contacts leading to transmission events within a population (Clay et al., 2009; Woolhouse et al., 1997). This type of heterogeneity can give rise to infectious disease superspreaders (Lloyd‐Smith et al., 2005), supershedders (Chase‐Topping et al., 2008; Lass et al., 2013), supermovers, and supersusceptibles (Craft, 2015). The growing evidence and descriptions of social networks in many animals suggests that behavior is a critical component of their ecology and natural history and thus provides a great opportunity for animal managers to begin accounting for behavioral heterogeneity and incorporating it into the study and management of free‐ranging vertebrates (Conrad et al., 2011).

Behavioral heterogeneities among individuals can emerge during competition for resources, for example, delimiting “home” areas, attracting mates, or finding food. Some animals exhibit social behaviors, especially when foraging for food (Evans et al., 2018; Karplus et al., 2007; Kurvers et al., 2014). As individuals gather to feed, a network of social connectivity forms. In such networks, individual foraging variation can drive social organization (Methion & Díaz López, 2020), for example, a few individuals may be unusually bold or aggressive (Evans et al., 2018; Klefoth et al., 2013). Studies of foraging behaviors in social animals have suggested individuals may be more or less aggressive for resources (Aplin et al., 2015; Huntingford et al., 2010; Methion & Díaz López, 2020; Tóth et al., 2017). Boldness or aggression is often associated with greater tendency to explore novel patches of food despite higher risk of predation (Evans et al., 2018). Understanding how foraging can drive social behavior and connectivity in non‐native habitats may be of particular importance for controlling invasive species (Koehn, 2004; Kulhanek et al., 2011; Vilizzi et al., 2015).

One of the world's most invasive species is the common carp (*Cyprinus carpio)*, a large‐bodied cypriniform fish (Koehn, 2004; Kulhanek et al., 2011; Vilizzi et al., 2015). Common carp, herein “carp,” have long life spans, high fecundity, a general lack of predators as adults, and the ability to use a wide range of habitats and food. As an invasive species, carp have contributed to extensive declines in the abundance and diversity of macrophytes, waterfowl, and amphibians and can also reduce recreational lake use by increasing water turbidity and nutrient concentrations (Bajer et al., 2016; Haas et al., 2007; Kloskowski, 2011; Kulhanek et al., 2011). Carp are highly social and are known for forming large aggregations during seasonal migrations, spawning, and overwintering. Carp's social foraging behaviors are of particular interest to ecologists and managers because they not only occur naturally but can also be manipulated via training/conditioning techniques. Laboratory experiments have shown that carp apply social learning strategies where naïve individuals can learn locations of food resources by observing trained individuals (Zion et al., 2007). Field experiments in lakes showed that carp can learn locations of novel bait sites in only a few days and consistently return to them at night from remote (~500 m) “home” sites occupied during the day (Bajer et al., 2010; Ghosal et al., 2018). Similar learning behaviors were reported from aquaculture operations where carp quickly learned to exploit automated feeders established for more valuable commercial species (Zion et al., 2012). Carp can also respond to stressful stimuli associated with bait and learn hook avoidance while foraging in systems frequented by anglers (Klefoth et al., 2013).

The fact that carp can be conditioned to form large feeding aggregations suggests that novel management techniques could be developed for this species. For example, “box nets” that lay on the bottom and whose sides can be quickly lifted above water might be especially effective for removing carp that gather at bait (P. G. Bajer, unpublished data), especially when carp aggregate in large numbers synchronously. However, carp's response to baiting may not be equally shared among individuals. Laboratory experiments suggest that carp display personality traits (i.e., bold‐shy spectrum), in which bold individuals may be more likely to “discover” novel food patches and outcompete shy individuals for access to them (Górecki et al., 2019; Huntingford et al., 2010). This might result in high foraging inequality (i.e., 20/80 rule), which would reduce the effectiveness of management through physical removal. High site fidelity where carp are unlikely to aggregate at the bait from distant areas, or asynchronous feeding where some individuals feed at different times to avoid competition might have similar effects. Managers could apply measures to counteract these effects. For example, increasing the number of baited sites could be used to increase access for shy and more sedentary individuals, while manipulating the temporal availability of the bait (e.g., through remotely controlled feeders) might force the carp to aggregate at the bait at the same time. These key aspects of carp's social feeding behaviors such as feeding equality, synchronicity, and site fidelity have not been adequately described in natural environments.

To address these aspects of carp foraging aggregations, we used passive integrated transponder (PIT) tags to observe carp visits to baited sites. Using this high‐resolution temporal and spatial data, we investigated: (1) how carp respond to baiting at multiple sites in a natural lake; (2) patterns of co‐visitation between feeding sites; (3) feeding bout group size and duration; (3) the most frequently detected feeders (hereafter “superfeeders”); and (4) the heterogeneity and connectivity of carp social networks at foraging sites. Overall, we hypothesized that carp's foraging behaviors may follow the 20/80 rule that has been documented for other social and foraging behaviors and that the carp will exhibit considerable site fidelity with closely located baiting sites having most co‐visits. By better documenting carp foraging behaviors in response to baiting, we provide foundational data for the development of efficient, cost‐effective removal methods for these invasive fish.

## 2 MATERIALS AND METHODS

### 2.1 Study location and unique identifiers

**FIGURE 1:**
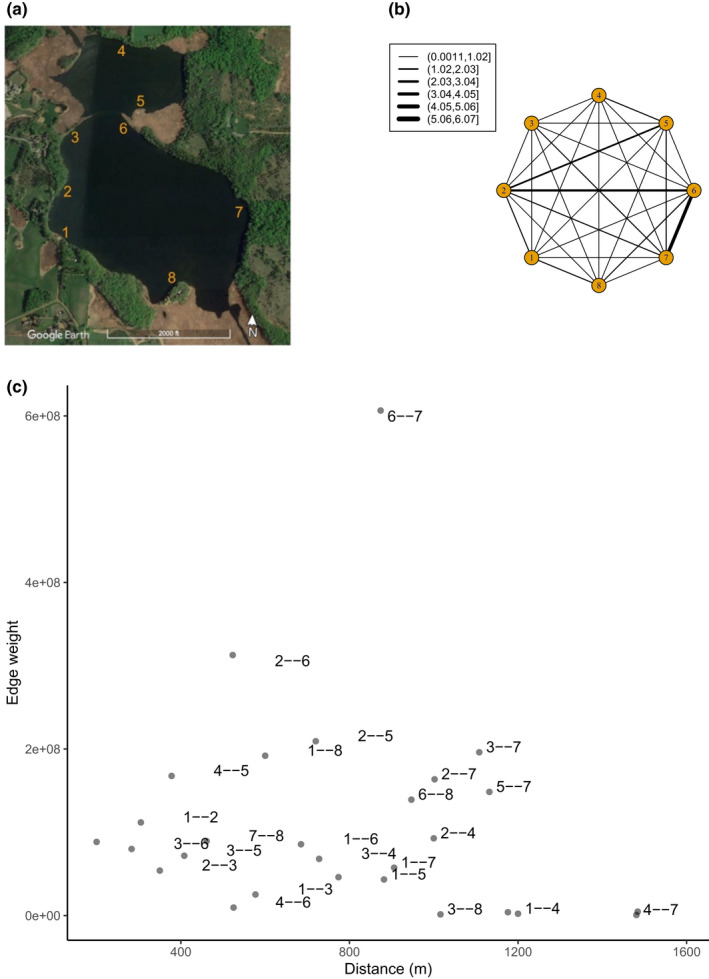
(a) Study sites in Parley Lake. In 2019, Sites 1 and 3 were established for monitoring. Satellite imagery was obtained from Google Maps. In 2020, six additional sites were added for a total of eight sites in the 2020 season. (b) Bipartite one‐mode projection network depicting the relative scale of shared detection of fish between different sites in Parley Lake in 2020. An edge between two sites indicates that at least one fish was detected at both those sites. Thicker edges indicate a greater extent of shared detections between sites. For depiction, edge weights were scaled by a factor of 10^8^. For example, the heaviest edge weight between Sites 6 and 7 corresponds to 6.065 × 10^8^ mutual detections. (c) Plot of distance between Sites and their calculated edge weights. Individual points correspond to a unique edge between Sites (e.g., “5 ‐ ‐ 8” corresponds to the edge between Sites 5 and 8). To avoid overlapping text, text labels are jiggered and not all edge labels are shown

We conducted the study between July 23 and August 16, 2019, and June 23 and July 23, 2020, in Parley Lake (44°52′51.8″N 93°43′54.1″W) located 52 km west of Minneapolis, MN. Parley is a moderately sized (105 ha), shallow (max depth 6 m) lake (Figure 1a) split into two basins: the upper basin is smaller, shallower, and has patches of aquatic vegetation, while the lower portion is larger and consists of more open water. Approximately 300 m upstream of Parley Lake, there is large (~40 ha) shallow marsh called Mud Lake. The marsh is permanently connected with Parley Lake and the carp may move between the two systems throughout the year. However, carp were unable to move out of the Parley Lake‐Mud Lake system because of a physical barrier just downstream of Mud Lake (44°54′05.3″N 93°43′59.1″W). There is also another physical carp barrier immediately upstream of Parley Lake (between Parley Lake and Lunsten Lake) preventing carp movement in that direction. In summer 2019, the Parley system was inhabited by approximately 18,000 carp (Carp Solutions, 2021).

We conducted boat electrofishing surveys around the perimeter of the entire lake (June 8–12, 2019) and implanted 344 carp with 12 mm‐HDX passive integrated transponder (PIT) tags (Oregon RFID). Of the 344 individuals, 102 were female, 193 male, and 49 were of undetermined sex. The average length was 615.3 ± 131.6 mm (*n* = 344). No carp were tagged in Mud Lake because that system is difficult to navigate with a boat.

In 2019, two study sites were selected along the western shore of Parley Lake (Sites 1 and 3 in Figure 1a). Both sites were located ~5 m from shore and were ~1 m deep. At each site, we installed a Multi‐Antenna HDX PIT reader (Oregon RFID) connected to three, 1m diameter pass‐over PIT antennas (12 gauge copper wire supported by a hula hoop frame) placed on the bottom of the lake, parallel to shore and spaced 5 m from one another. Each reader was powered by 12 V deep cycle batteries charged by a solar panel array (GrapeSolar Eugene). Default scanning parameters were used resulting in 10 scans per second, divided across three antennas per site, a total of 3.33 scans/second/antenna. We used the default PIT reader settings and checked tuning, read range, and scanning function daily. Nylon mesh bags (70 L, 4 mm mesh size) were placed on the lake bottom in the center of each antenna. Depending on the phase of the experiment (see below), the bags would be filled with uncooked cracked corn. Importantly, native fish species found in this geographic region do not appear to be attracted to cracked corn (Bajer et al., 2010). Carp could pull corn through the mesh, but uneaten corn remained, allowing the rate of consumption to be monitored. Six additional study sites were added during the 2020 experiment for a total of eight sites (Figure 1a). In 2020, each site was designed as in 2019, but with only a single central pass‐over PIT antenna per site instead of three per site.

### 2.2 Study design

The study proceeded in two phases annually: prebaiting and baiting. In the prebaiting phase, we collected detections in the absence of bait (July 23–July 30, 2019, and June 23–July 3, 2020). Prebaiting was followed immediately by a baiting phase between July 30 and August 16, 2019, and July 3 and July 23, 2020, respectively. During the baiting phase, we placed cracked corn (>55 kg per antenna) in the mesh bags daily, usually between 10:00 and 14:00 h to avoid peak carp activity. After the 2019 experiment concluded, carp managers used box nets and seines to remove approximately 5935 carp total (2333 carp removed in late summer of 2019 and 3602 removed in winter of 2020). Among those, 69 PIT‐tagged carp were captured. All PIT‐tagged carp were released except for 5, which were euthanized. For clarity of presentation of the data detected from the most antennas, we focused primarily on the 2020 season and have provided the 2019 results in the Appendix S1.

### 2.3 Analysis

#### 2.3.1 Co‐visitation between sites

We generated a bipartite network of co‐visitation with the eight sites in 2020 serving as one set of nodes and individual carp serving as the second set of nodes. An edgelist was formed with each row including the fish ID and detection site. Therefore, the existence of an edge between two nodes (sites) in the one‐mode projection network indicates that at least one fish visited both sites. Edges were weighted to reflect the relative intensity of co‐visitation between sites of all tagged fish in the population.

#### 2.3.2 Gaussian mixture model analysis

**FIGURE 2:**
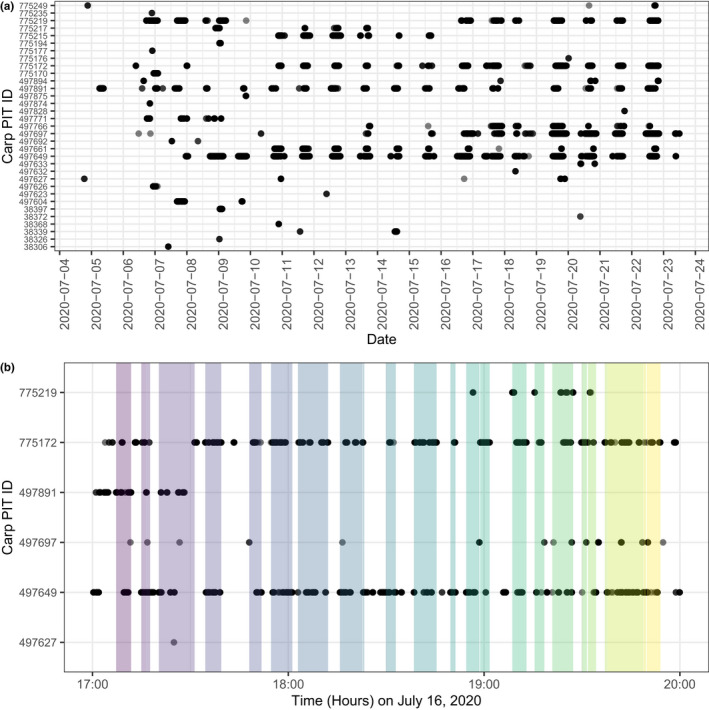
Depiction of spatio‐temporal data collected with PIT tag readers. (a) Individual detections of carp for the baiting period at Site 1 in the 2020 season. Time is on the *x*‐axis and individual carp are on the *y*‐axis. (b) Detailed version of carp detected for a span of three hours on the evening of June 16, 2020, at Site 1. Presumptive co‐feeding events as classified by the GMM analysis are highlighted with different shadings of color

We created unipartite social networks of PIT‐tagged carp at feeding sites using a Gaussian mixture model (GMM) approach (Psorakis et al., 2012, 2015). Rather than establishing an arbitrary time threshold to detect social associations, this approach identifies high‐density activity periods in order to determine group associations (Figure 2b). For example, a GMM approach has been used to look at: foraging associations and social phenotypes of wild great tits, *Parus major* (Aplin et al., 2015); social dominance and initiation of foraging events in black‐capped chickadees, *Poecile atricapillus* (Evans et al., 2018), and the effects of provisioning on social behavior of tiger sharks (*Galeocerdo cuvier*) at a dive tourism location (Jacoby et al., 2021).

These analyses were carried out in the asnipe package using the gmmevents function in R to identify temporal group associations from the PIT tag data (Farine, 2019). This method resulted in a group‐by‐individual matrix and a list of the duration of each observed bout. From this output, we then calculated the mean group size per bout and the mean bout duration. We then created a network from the resulting group‐by‐individual matrix using the get_network function, which creates edges between individuals in the network based on whether they co‐occur with other individuals in a bout captured in the group‐by‐individual matrix (Farine, 2019). To calculate edge weights in the network, we used the half‐weight association index, which, unlike the simple ratio index, can account for missing observations of individuals or groups (Hoppitt & Farine, 2018).

#### 2.3.3 Superfeeders, site fidelity, and network metrics

Here, we analyzed carp feeding behavior in accordance with the 20/80 rule, wherein 20% of individuals in a population are responsible for 80% of the behavior of interest (Clay et al., 2009; Woolhouse et al., 1997). To identify potential superfeeders, we created Lorenz curves by plotting individual foraging rank against total cumulative detections (Crall et al., 2018). We classified those carp accounting for 80% of cumulative total detections in each season as superfeeders. We then calculated the Gini coefficient, which serves a measure of inequality or dispersion in a frequency distribution. From a Lorenz curve, the Gini coefficient corresponds to the area between the line of perfect equality and the observed Lorenz curve divided by the total area under the line of perfect equality (Crall et al., 2018). A Gini coefficient close to zero represents equality (all individuals were detected foraging the same number of times), while a Gini coefficient close to one corresponds to strong inequality (one or a few individuals were detected foraging the most).

To examine relative site fidelity for each individual, we divided the number of detections at the most visited site by the total number of detections and then compared these fractions using a Welch two sample t test. In addition, we compared the body length of superfeeders vs. non‐superfeeders using a Welch two sample t test and conducted a logistic regression using the glm function in R (binomial family) to assess how the likelihood of being a superfeeder depends on body length. We also assessed whether superfeeders were more or less likely to be detected earlier in the baiting period by comparing date of first detection for superfeeders vs. non‐superfeeders.

To examine the network metrics of superfeeders vs. non‐superfeeders, we analyzed unweighted degree (to measure the number of interactions with different conspecifics), weighted degree (to measure the combined number and intensity of those interactions), and betweenness (to identify individuals that have an outsized role in connecting disparate parts of the network) (Aplin et al., 2015). Other studies analyzing social foraging behavior with GMM have commonly used these metrics, and these are also widely used network metrics for understanding social behavior in wildlife more generally (Aplin et al., 2015; Evans et al., 2018; White et al., 2017).

We created and analyzed networks using the igraph and ggnetwork packages (Briatte, 2020; Csardi & Nepusz, 2006). All other plots were generated using base R or the ggplot2 package (Wickham, 2016). Analyses were conducted in R (version 4.0.1).

### 2.4 Video observations

Video recordings were included as a visual reference of carp feeding aggregations at corn baited feedings sites in a nearby lake in New Brighton, Minnesota, USA (Long Lake: 69.8 hectare, maximum depth 8 m, ~45 km NE of Parley Lake) (Video S1). Videos were recorded using a Micro plus 5 camera (Aqua‐Vu), placed ~1 m from the bait. Videos were recorded between 16:00 and 22:00 in 2018 (26, 28 September and 1, 2, 7 October). We have included one video from 26 September 2018 to help visualize carp group feeding at a bait (Video S1).

## 3 RESULTS

### 3.1 Detections

In total, there were 96,030 and 616,667 detections of PIT‐tagged carp during the 2019 and 2020 seasons, respectively. The antenna detection range was ~30 cm and always greater than 21 cm.

#### 3.1.1 Prebaiting

Few individuals were detected during the prebaiting periods. During the 2019 prebaiting period (7 days: July 23–July 30), there were only 184 detections of 11 unique carp (Figure S1A). Likewise, during the 2020 prebaiting period (11 days: June 23–July 3), there were 74 total detections of 12 unique carp (Figure S1B).

#### 3.1.2 Baiting

Carp responded quickly to baiting. During the 2019 baiting period (17 days: July 30‐August 16), 107 unique carp were detected at the two sites (Sites 1 and 3; Figure 2a). Only 38 individual carp were detected at both Sites 1 and 3. Seventy were detected at Site 1, while 75 were detected at Site 3. During the 2020 baiting period (20 days: July 3–July 23, 2020), 133 unique fish were detected across the eight sites. Uniquely tagged carp per site ranged from 33 (Site 1) to 50 (Site 2) (Table S1). Up to 31% of carp per site had “site fidelity” to that particular site, but most carp were detected at multiple feeding sites (Table S1, Figure 2b). Annual participation by individuals varied: 57 carp were detected only during the 2019 baiting period (of those, five were removed), 83 carp were detected only during the 2020 baiting period, and 50 carp were detected during the baiting periods of both years.

The highest number of uniquely detected carp per day occurred five and four days from the start of bating in 2019 and 2020, respectively (Figure S1). In both years, carp were most frequently detected at the feeding sites starting at dusk and into the morning with few detections during peak daylight hours (Appendix S2).

### 3.2 Co‐visiting between sites

The distance between our feeding sites ranged from ~200 m (Sites 5 and 6) to ~1485 m (Sites 4 and 8) (Figure 1a). There was some evidence that feeding at multiple sites across the lake was constrained by basin geography. For example, no fish were detected at both Site 4 (the northmost site in the upper basin) and Sites 1, 7, and 8 (the southernmost sites in the lower basin) (Figure 1b). However, distance did not appear to be the only covariate for explaining co‐visitation rates between sites. Despite being four‐fold further apart (~870 m apart) than Sites 5 and 6, Sites 6 and 7 had the highest frequency of co‐occurring fish, and a corresponding edge weight in the bipartite network that was 6.9 times more dense than the edge corresponding to Sites 5 and 6 (Figure 1b,c).

### 3.3 Feeding bout group size and duration

**FIGURE 3:**
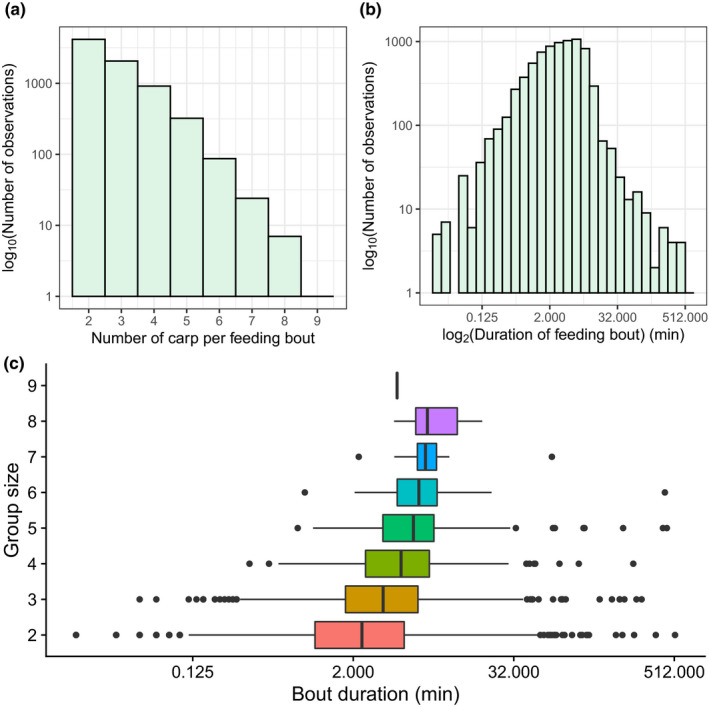
Feeding bout (a) group size, (b) duration, and (c) correlation between group size and duration for 2020. (a, b) Histograms of group size and duration based on GMM analysis of spatio‐temporal PIT tag data. (c) Box plots of bout duration, grouped by detected group size based on GMM analysis. Note that the *y*‐axes for (a) and (b) are on a log10 scale, and the *x*‐axes for (b) and (c) are on a log2 scale for the purposes of visualization

Using a GMM approach, we calculated the group sizes and durations of the estimated feeding bouts during the corn baiting period in both 2019 and 2020 for individual sites and across the entire lake (Figures 2b and 3a, Table S2). For 2020, the detected feeding bouts at an individual site ranged in size from two to nine PIT‐tagged carp, but this distribution was heavily right‐skewed with most feeding bouts including only two to four PIT‐tagged carp (Figure 3a). While feeding bouts lasted on average 5.11 min with a median duration of 3.07 min, there were a few instances of feeding bouts lasting upwards of 30 min to over 8 h (Figure 3b). The majority of the longer associations were among smaller groups of PIT‐tagged carp (i.e., two to six individuals) (Figure 3c). Results from 2019 were similarly heavily right‐skewed, but with longer bouts (Figure S3A) and smaller groups (Figure S3C). One key difference was that the maximum detected group size in 2019 reached 16 individuals when combining all PIT tag antennas for a given site. In 2019, feeding bouts had an average duration of 6.13 min, a median duration of 4.5 min, and a maximum duration of 238 min (~4 h) (Figure S3B, Table S2). On average, in the 2020 baiting period, there were 2395 feeding bouts detected per day (ranging from 60 on the first day of baiting to 7831 on the seventh day of baiting).

### 3.4 Superfeeders and site fidelity

**FIGURE 4:**
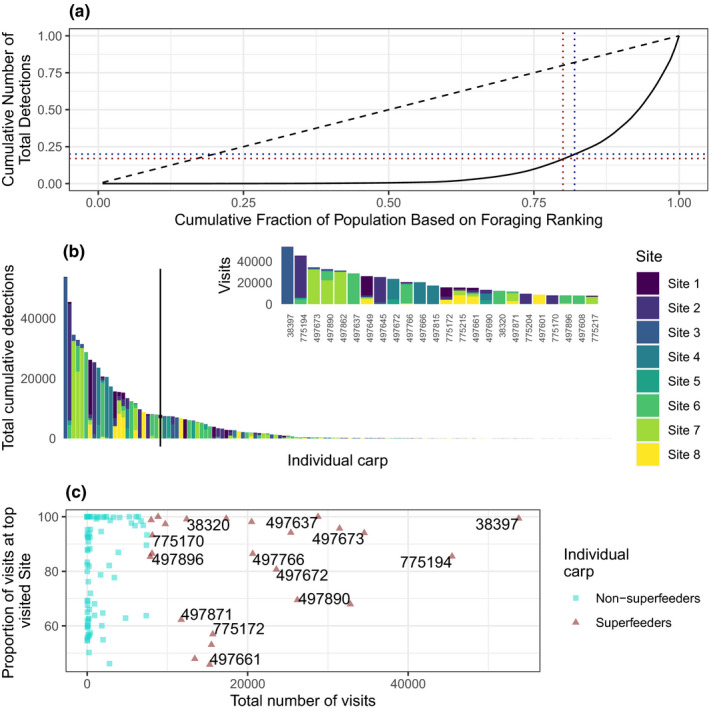
Carp superfeeding behavior in Parley Lake during the 2020 season. (a) Lorenz curve of foraging rank vs. cumulative number of total detections. The blue horizontal dashed line corresponds to 20% of total detections and the red vertical dashed line corresponds to 80% foraging rank. (b) The number of unique detections that individual carp were detected at each site across Lake Parley in the 2020 season. The vertical line delimits carp accounting for 80% of total cumulative detections, which are highlighted in the inset. (c) The relative site fidelity of superfeeders vs. non‐superfeeders as total number of unique daily visits versus the proportion of visits to the most‐frequently visited site per individual

In 2020, the top 20% of feeders accounted for 83% of the cumulative number of detections (Figure 4a, red‐dashed lines), while 80% of the cumulative number of detections were performed by the top 18% of feeders (Figure 4a, blue‐dashed lines). In 2019, the top 20% of feeders accounted for 66% of the cumulative number of detections (Figure S4A, red‐dashed lines), while 80% of the cumulative number of detections were accounted for by the top 29% of feeders (Figure S4A, blue‐dashed lines). Based on these definitions of superfeeders, there were 24 superfeeders in 2020 and 32 superfeeders in 2019. Across years, 9 fish recurred as superfeeders: 38% of superfeeders in 2020 and 28% of superfeeders in 2019.

**FIGURE 5:**
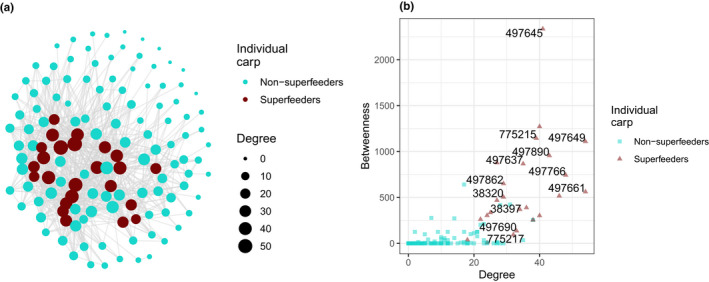
Social network analysis of carp co‐feeding in 2020 in Parley Lake. (a) Social network depicting individual carp as nodes and mutual detection of carp in GMM feeding bouts as edges. Node size corresponds to degree. (b) A scatterplot showing the relationship between degree and betweenness scores of individual PIT‐tagged carp. The labels of individual carp are jittered and removed in case of overlap. Throughout both panels, superfeeders are shown in maroon and non‐superfeeders are shown in sky blue

The Gini coefficient for foraging inequality was 0.79 in 2020 and 0.66 in 2019. In 2020, superfeeders were not more or less likely (83.2 ± 18.2% SD) than other fish (86.8 ± 16.9% SD) to visit their top visited site relative to other sites (Welch Two Sample *t*‐test, *t* = −0.88, df = 32.22, *p*‐value = .39) (Figure 4c). Despite having fewer detection sites, the same pattern held true in 2019: 96.7 ± 7.9% SD of visits were to the top visited site for superfeeders vs. 95.6 ± 10.0% SD for non‐superfeeders (Welch two‐sample *t* test, *t* = 0.60, df = 72.81, *p*‐value = .55) (Figure S4C). In 2020, we found that superfeeders were 11.2 cm longer than non‐superfeeders (Figure S5): mean length for superfeeders was 69.8 ± 55.5 cm vs. 58.6 ± 144.7 cm for non‐superfeeders (Welch two‐sample *t* test, *t* = 6.5461, df = 118.27, *p*‐value = 1.6 × 10^−9^). Based on a logistic regression, the difference in the log‐odds for an increase in 1 mm length was 0.011 or a 1.1% increase in the odds of being a superfeeder for every increase in length of 1 mm (Figure 5b, Table S3). We found similar superfeeding and site fidelity trends in 2019 (Figures S4 and S6).

To compare network position with the induction of feeding behavior, we compared the day at first detection for superfeeders against other carps. In 2020, the median date of first detection for supersfeeders was on June 4, 2020, or 0.58 days after the start of baiting compared to June 6, 2020 or 2.59 days after baiting for non‐superfeeders (Figure S7). In 2019, the median date of first detection for superfeeders was on August 1, 2019, or 1.79 days after the start of baiting compared to August 3, 2019, or 3.17 days after baiting for non‐superfeeders (Figure S7).

### 3.5 Co‐feeding network heterogeneity and connectivity

We derived a social contact network, with group membership indicated by GMM analysis, based on co‐feeding events during 2020 (Figure 5a). On average, superfeeders had a higher degree (34.9 ± 9.7 SD) compared to non‐superfeeders (10.6 ± 9.7 SD) (Welch two‐sample *t* test, *t* = 11.15, df = 32.43, *p*‐value = 1.2 × 10^–12^); a higher weighted degree (1.25 ± 0.29 SD) compared to non‐superfeeders (0.26 ± 0.30) (Welch two‐sample *t* test, *t* = 15.03, df = 35.03, *p*‐value = 2.2 × 10^–16^); and a higher betweenness (604.2 ± 513.8 SD) compared to non‐superfeeders (39.8 ± 98.4 SD) (Welch two‐sample *t* test, *t* = 5.35, df = 23.37, *p*‐value = 1.8 × 10^–5^) (Figure 5b).

### 3.6 Video observations

Video recordings showed multiple carp within close proximity to the bait bag, of which a few were foraging on the bait for several seconds then slowly moving away while others took their place. No carp were observed aggressively bumping or nipping other carp, a common sight for other fish species at baited sites.

## 4 DISCUSSION

In this study, we induced the foraging behavior of wild carp by using corn (bait) to foster aggregations and observations of social carp behavior in a natural lake. Carp responded quickly, forming aggregations that were most active from dusk to dawn (Figures S1 and S2). The addition of more sites and reduction of antennas per site from 2019 to 2020 resulted in fewer unique detections per site (Table S1), more total detections, and possibly fostered foraging behavior with the abundance of food. Collectively, our results indicated that carp foraging is social, easily induced by species‐specific bait, dominated by large‐bodied individuals, and potentially predictable, suggesting that carp social behavior and individual preference should be considered in the management of one of the world's most recognizable and invasive fish species.

Our observations suggest that carp feeding aggregations are large, dynamic, and likely divided into smaller social subunits. GMM analysis of carp visits to baiting sites classified feeding bouts as short, with frequent turnover of small groups (Figure 2b); these findings were supported by direct behavioral observations (Video S1). This type of foraging behavior supports “scramble competition” described in laboratory carp by Huntingford et al. (2010) and further suggests that carp are not aggressive foragers. Rather, carp appeared to have open access to an unlimited food source (i.e., corn). Short feeding bouts within a known large feeding aggregation may be related to: a social hierarchy among individuals (Ward et al., 2006), smaller social subunits (reviewed in Krause et al., 2007), feeding mode, or limitations of the detection system. For example, Cypriniforms, like carp, have oral jaws which remove “food” (detritus, invertebrates, and seeds like cracked corn) from the substrate to be sorted using a unique muscular palatal organ (Hernandez & Cohen, 2019). Unwanted particles are discarded and desired food is passed to pharyngeal teeth for further processing (reviewed in Gidmark et al., 2012). The cyclic process could be observed as carp were swimming away from the bait actively chewing with pharyngeal jaws, with debris and small food particles spilling out of the mouth and operculum (Video S1). The result was many actively swimming carp, a few feeders, and a water column spotted with suspended corn. Individual carp cycled in and out of the feeding site, in a process which likely tested the detection tracking limitations of PIT technology (Gibbons & Andrews, 2004).

We found that carp displayed individual heterogeneity and strong inequality in foraging behavior, where a small number of individuals accounted for the majority of cumulative detections (Figure 4). These superfeeders had a higher number of contacts in the network (i.e., degree, weighted degree, Figure 5b) and were more likely to connect disparate parts of the co‐feeding network (i.e., betweenness, Figure 5b). These observations are consistent with the “20/80 rule” and the so‐called “small world” network topology where a few long‐distant edges in the network can increase transmission of information and disease (Watts & Strogatz, 1998). From a management perspective, superfeeders may be the key to removal strategies, since they are contributing more to social learning via their network position and possibly encouraging other fish to feed at baited sites (e.g., “leaders” in cod aquaculture (Björnsson et al., 2018)). Indeed, superfeeders in this study were, on average, detected earlier at baiting sites in both 2019 and 2020 seasons (Figure S7). Individual behavior of fish can be influenced by others and in response to management efforts, leading to a nonrandom or uneven distribution of behavior types. For example, Monk et al. (2021) found that sustained angling pressure on northern pike *Esox lucius* resulted in “small, inactive, shy, and difficult‐to‐capture fish,” not purely by natural selection suggesting that removal strategies impact fish populations (such as through behavior and growth). The resulting harder‐to‐catch fish and likely diminished management success suggest the superfeeders could be important and warrant more study.

In our study, superfeeders were substantially larger than other carp (Figure S5). In many fishes, length is an indicator of social hierarchy, age, and possibly also temperament (Froese & Pauly, 2021; Huntingford et al., 2010; Ward et al., 2006) where the largest tend to dominate. Björnsson et al. (2018) hypothesized that the largest fish might be present at the bait most often and consume most of it due to their higher caloric requirements and higher energy costs associated with active foraging. Our observations are in line with other studies that have found that body size could predict variation in contact behavior; for example, in a study of the contact behavior of deer mice (*Peromyscus maniculatu)s*, larger mice were more connected and had more contacts than smaller mice (Clay et al., 2009). The larger, more connected individuals may play a critical role in aggregating many carp into a small area; a scenario ripe for management via removal, biocontrol, “bait and switch” toxic food pellet strategy (Hundt et al., 2020; Poole et al., 2018), or a novel method. For example, if using a species‐specific targeted, transmissible biocontrol strategy (e.g., koi herpes virus, McColl et al., 2018; Padhi et al., 2019; Thresher et al., 2018), targeting large, superfeeders could help improve the reach of such biocontrol through greater contacts with other individuals and disparate parts of the lake. Although other traits like sex and health status (e.g., diseased vs. healthy, Croft et al., 2011) may be correlated with network position, we were unable to test for other traits of wild carp. Future studies could focus more on behavior, movement, and traits of these superfeeders and determine if removal or retention of these individuals is more beneficial.

There are several abiotic and biotic factors with this method that may serve as barriers to carp management. Beyond individual behavioral heterogeneity, carp display ecological plasticity, resulting in regional or possibly hyperlocal management planning. Our study was designed for central Minnesota; therefore, carp managers in different regions will need to examine: bait choice (species specificity, environmental impact), location constraints (e.g., water depth, access, plant cover, number of sites), detection system (PIT, acoustic telemetry), number of fish to tag, and removal/management system (nets, biocontrol, cages). For example, if corn was used as an attractant in a central Europe, many species of larger bodied cyprinid fishes (e.g., Tench (*Tinca tinca*) and bream (*Abramis brama*)), would also aggregate, leading to possible by‐catch. Since there are not universal carp‐specific baits yet, managers should explore other stimuli to induce carp aggregations and determine the utility of different removal tools.

An important limitation of this study is that a relatively small proportion of the population was tagged (344 tagged carp in a population of ~18,000). While it is difficult to speculate how that might have influenced the results of our experiment, some observations can be drawn. First, despite the fact that only ~1 in 60 carp in the lake was tagged, we did observe considerable differences among individuals (e.g., presence of superfeeders, dominance of large‐bodied individuals). Had we tagged a greater proportion of the population (or had we tagged all carp), it is very likely that even more dramatic differences among individuals would be detected (e.g., ultra‐feeders, ultra‐dominants of baited sites). Overall, this suggests that in large populations of carp there is a very wide range of behaviors exhibited by individuals. However, the fact that we tagged only ~1 in 60 carp has important limitations. Specifically, our study was not designed to monitor small aggregations of carp at the bait (i.e., groups less than ~60 individuals), because such aggregations could occur while no tags were detected. Our study focused on detecting large social aggregations, which are of primary importance in developing new management schemes.

It was also somewhat surprising that we did not detect a greater proportion of the tagged carp at the bait in 2020 (e.g., 133 unique carp were detected across all eight baited sites in 2020). Earlier experiments (Bajer et al., 2010), where approximately 70% of radiotagged carp were attracted to a single baited site in a small (35 ha) lake, would suggest that nearly all tagged carp should have been detected across the eight baited sites in this experiment. We hypothesize that this was attributable to a few factors. First, a significant portion of the carp population might have inhabited the large marsh upstream of Parley Lake (Mud Lake). Because that system is largely inaccessible by boat, we were unable to place baiting stations there. Further, some carp have likely lost their tags or perished due to natural causes throughout the study. PIT tag loss rates typically range between 10% and 20% however rates as high as 50% have also been reported (Musselman et al., 2017). We were unable to monitor the tag loss in our study, but in other experiments we observed approximately 15% PIT tag loss in common carp per year (P. G. Bajer; unpublished data).

Since size and timing of peak aggregation are critical to an optimized management strategies, future studies should further tease apart the role of superfeeders and connectivity of social subunits to aid in accurately predicting peak aggregation. Defining a relevant contact remains a universal challenge in wildlife studies, regardless of whether it is for understanding the transmission of social information or disease or for timing interventions for maximum impact. However, the process of obtaining network structure by discretizing a continuous observation stream is a nontrivial task (Psorakis et al., 2015). Here we used a GMM analysis approach (Figure 2b) which eliminates the need to predefine a biologically meaningful contact window and the assumption that the time window for interactions must be constant (Psorakis et al., 2015). However, looking at the raw datastreams themselves, some individuals are steadily present at feeding sites for longer periods (e.g., carp #775172 and #497649 in Figure 2b), without this activity being categorized as a continuous feeding bout by the GMM approach. Therefore, one open question is how much heterogeneity in detected feeding bout size matters for understanding the actual number of carp present in a feeding aggregation: for example, does a detected feeding bout size of two fish vs. six fish truly correlate with a three‐fold increase in total swarm size? Further resolution is critical to tease apart interrelationships of individuals; future studies should use new technology such as high‐resolution acoustic telemetry systems to track all movement of individuals, not just at feeding sites. Ultimately, carp sociality may be a key to allowing managers to manipulate and induce feeding aggregations and begin to reduce their negative ecological impact worldwide.

## CONFLICT OF INTEREST

Przemyslaw Bajer is founder and owner of Carp Solutions LLC, a company that focuses on carp management. This activity is overseen by the University of Minnesota in accordance with its conflict of interest policies.

## AUTHOR CONTRIBUTIONS

**Peter J. Hundt:** Conceptualization (equal); Investigation (equal); Methodology (equal); Writing – original draft (lead); Writing – review & editing (equal). **Lauren A. White:** Data curation (lead); Formal analysis (lead); Methodology (equal); Writing – review & editing (equal). **Meggan E. Craft:** Conceptualization (equal); Formal analysis (equal); Methodology (equal); Project administration (equal); Writing – review & editing (lead). **Przemyslaw G. Bajer:** Conceptualization (equal); Funding acquisition (lead); Methodology (equal); Project administration (lead); Writing – original draft (equal); Writing – review & editing (equal).

## Supporting information

Supplementary Material

## Data Availability

Data and R code is available at: https://github.com/whit1951/Carp/ and https://doi.org/10.5281/zenodo.6025248. Supplementary video recording available via the Data Repository for U of M (DRUM) https://doi.org/10.13020/m21j‐ww77 (Hundt et al., 2020).
