# Supplementary material for: Social associations in common carp (Cyprinus carpio): Insights from induced feeding aggregations for targeted management strategies
Source: Ecol Evol. 2022 Mar 7;12(3):e8666. doi: 10.1002/ece3.8666 (PMC8901867; doi:10.1002/ece3.8666)
Supplement: Supplementary file 1 — Supplementary Material [file ECE3-12-e8666-s001.docx]

# APPENDIX


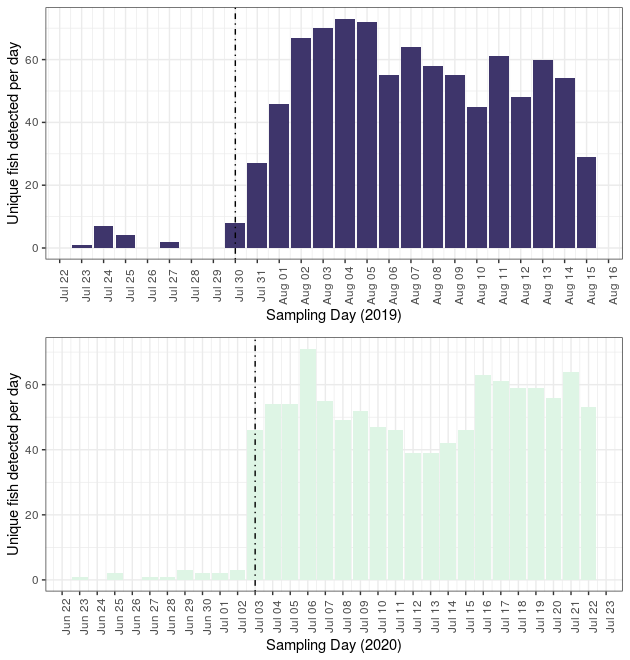


**Figure S1.** Total daily detections of unique carp at all sites in Parley Lake for the pre-baiting and baiting periods in **(A)** 2019 and **(B)** 2020. The dashed line indicates the start of corn-baiting, July 30, 2019 and July 3, 2020 respectively.


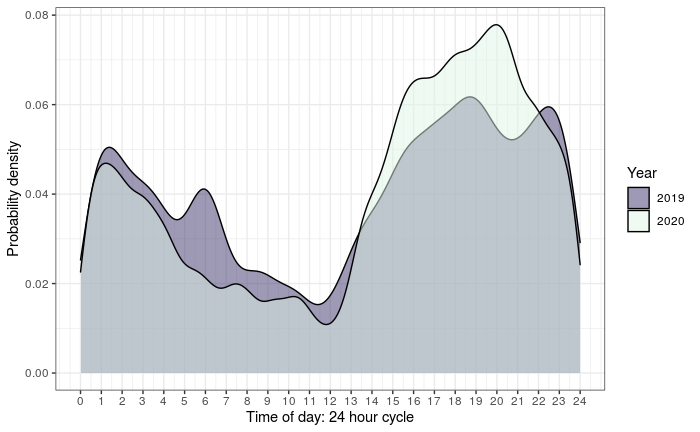


**Figure S2.** The probability density curve based on the total number of detections across a 24-hour cycle for the baiting period in both 2019 and 2020 in Parley Lake.


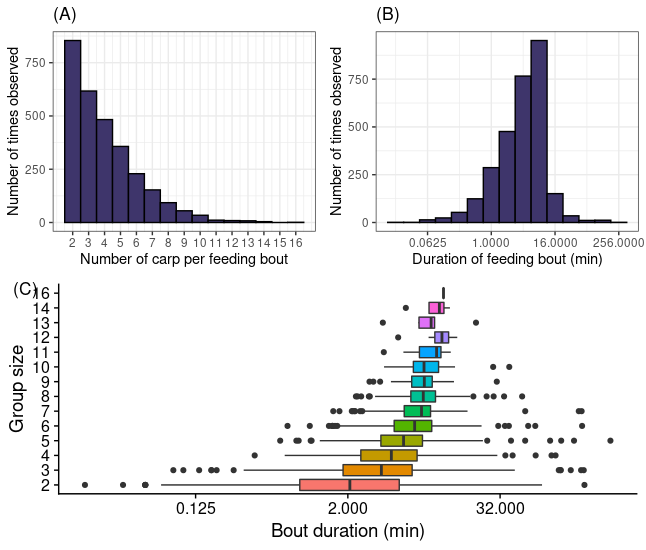


**Figure S3.** Feeding bout **(A)** group size, **(B)** duration, and **(C)** box plots of bout duration as a function of group size for 2019. **(A,B)** Histograms of group size and duration based on GMM analysis of spatio-temporal PIT tag data. **(C)** Covariation between bout duration and detected group size based on GMM analysis. Note that axes for B & C are on a log(2) scale for the purposes of visualization.


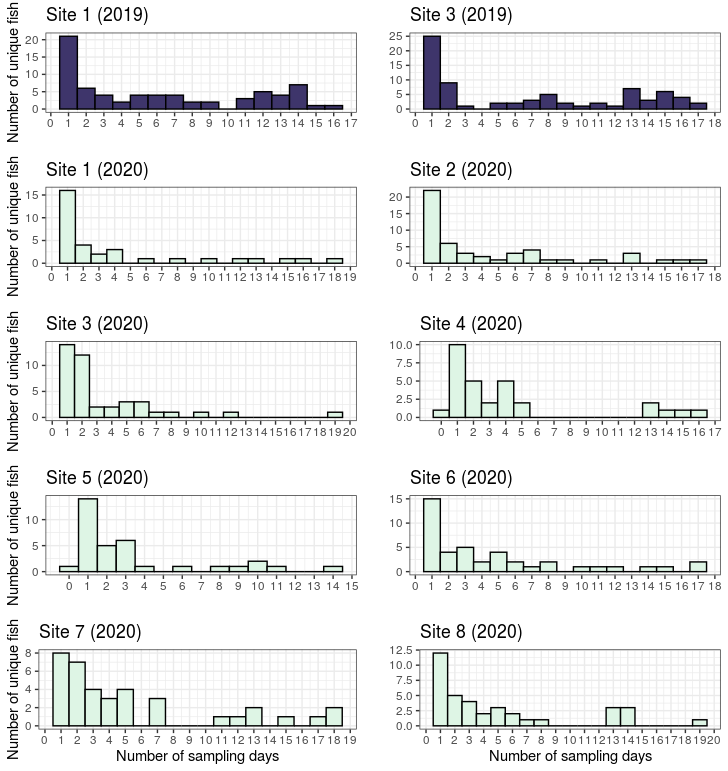


**Figure S4.** Additional histograms displaying the number of unique sampling days that individual fish visit each site during the baiting period for Sites 1 and 3 in 2019 and Sites 1-8 in 2020.


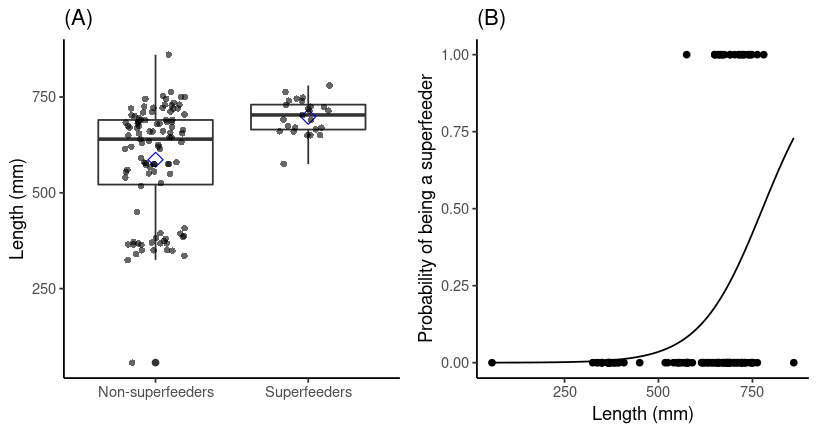


**Figure S5.** (A) Boxplot comparing measured length of superfeeders vs. non-superfeeders in 2020 at Parley Lake. Each boxplot displays the median (central line), the first and third quartiles (hinges at the 25th and 75th percentiles), and whiskers extending from the hinge to the largest or smallest value no further than 1.5 * inter-quartile range from the hinge. Individual data points are shown as jittered points. The mean is shown as a blue diamond. Mean length for superfeeders was 698.1 mm vs. 586.2 mm for non-superfeeders (Welch two sample t-test, t = 6.5461, df = 118.27, p-value = 1.6 x 10^-9^) (B) Measured length of fish vs. the probability of being a super feeder with a fitted logistic regression curve. Fitted values for logistic regression are in Appendix Table 3.


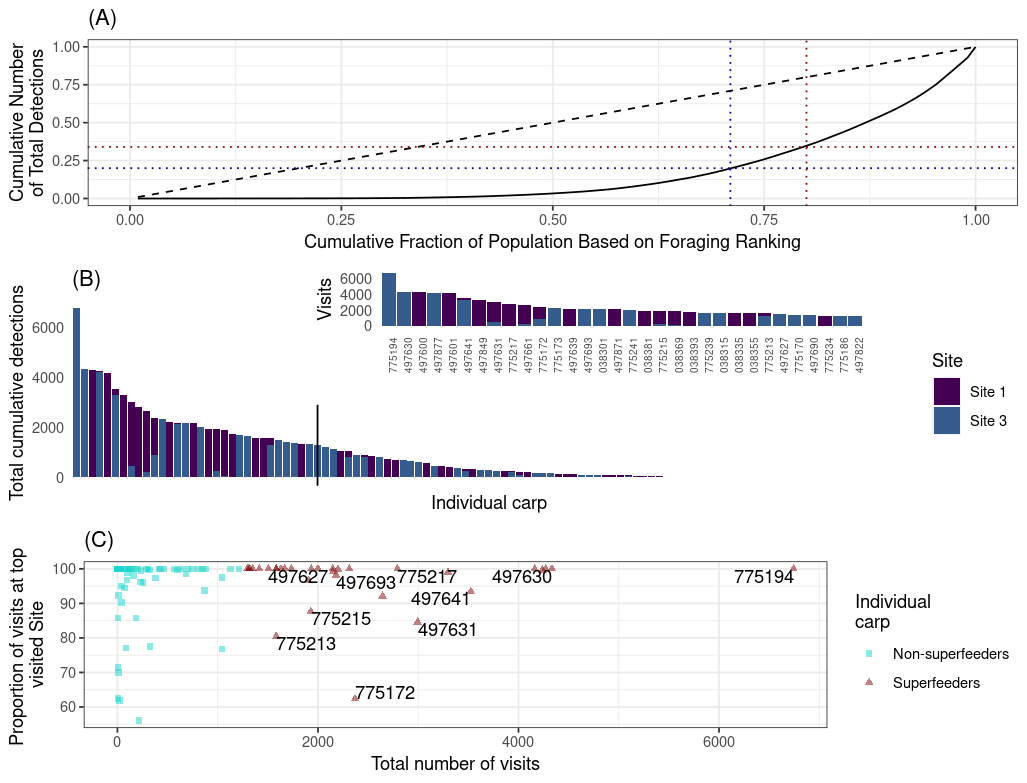


**Figure S6.** Carp superfeeding behavior in Parley Lake during the 2019 season. **(A)** Lorenz curve of foraging rank vs. cumulative number of total detections. The blue horizontal dashed line corresponds to 20% of total detections and the red vertical dashed line corresponds to 80% foraging rank. **(B)** A histogram of the number of unique detections that individual carp were detected at each site across Lake Parley in the 2019 season. The vertical line indicates the cut off between the carp accounting for 80% of total detections. The inset of this figure zooms in on the superfeeders to the left of the vertical line. **(B)** A scatterplot exploring relative site fidelity of superfeeders vs. non-superfeeders by comparing the total number of unique daily visits to proportion of visits at the most-frequently visited site per individual.


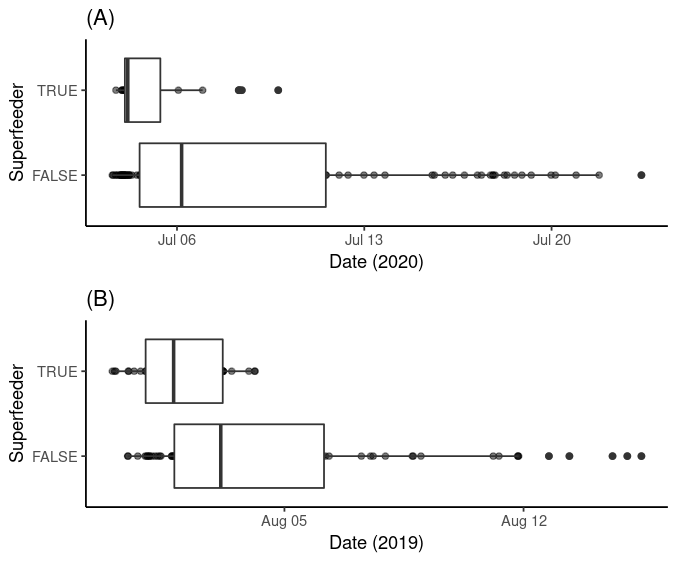


**Figure S7**. Box plots of date of first detection for superfeedering and non-superfeeding carp in **(A)** 2020 and **(B)** 2019 seasons.

**Table S1.**The number of individuals detected during the corn baiting period at only one site, the total number of unique individuals detected at each site, and the percent of unique detections per site Parley Lake in 2020.

| Site ID | Individuals unique to site | Total unique carp detected | Percent of unique detections |
| --- | --- | --- | --- |
| 1 | 4 | 33 | 12.12% |
| 2 | 2 | 50 | 4.00% |
| 3 | 9 | 41 | 21.95% |
| 4 | 4 | 30 | 13.33% |
| 5 | 0 | 34 | 0.00% |
| 6 | 13 | 42 | 30.95% |
| 7 | 4 | 37 | 10.82% |
| 8 | 10 | 37 | 27.03% |

**Table S2.** The average feeding bout during the baiting period in 2019 and 2020 as classified by Gaussian mixed models (GMM) for individual sites and Lake Parley as a whole.

|  | Mean (Median) Feeding bout in minutes | |
| --- | --- | --- |
|  | 2019 | 2020 |
| 1 | 6.35 (4.07) | 5.98 (4.1) |
| 2 | - | 4.60 (2.97 |
| 3 | 5.96 (4.78) | 4.76 (3.57) |
| 4 | - | 5.59 (4.8) |
| 5 | - | 8.77 (4.15) |
| 6 | - | 4.20 (3.03) |
| 7 | - | 3.49 (1.98) |
| 8 | - | 4.51 (3.58) |
| Whole Lake | 6.131 (4.52) | 5.11 (3.07) |

**Table S3. Logistic regression findings for the likelihood of being a superfeeder as a function of body length for the 2020 season.**

| **Coefficients** | **Estimate** | **Standard Error** | **z value** | **p-value** |
| --- | --- | --- | --- | --- |
| **Intercept** | **-9.102** | **2.691** | **-3.383** | **7.17 x 10-4** |
| **Length** | **0.011** | **0.004** | **2.937** | **0.003** |
